# Large Language Models in Healthcare and Medical Applications: A Review

**DOI:** 10.3390/bioengineering12060631

**Published:** 2025-06-10

**Authors:** Subhankar Maity, Manob Jyoti Saikia

**Affiliations:** 1 Biomedical Sensors & Systems Lab, University of Memphis, Memphis, TN 38152, USA; 2 Electrical and Computer Engineering Department, University of Memphis, Memphis, TN 38152, USA

**Keywords:** healthcare, large language models, LLMs, medical, patient, review

## Abstract

This paper provides a systematic and in-depth examination of large language models (LLMs) in the healthcare domain, addressing their significant potential to transform medical practice through advanced natural language processing capabilities. Current implementations demonstrate LLMs’ promising applications across clinical decision support, medical education, diagnostics, and patient care, while highlighting critical challenges in privacy, ethical deployment, and factual accuracy that require resolution for responsible integration into healthcare systems. This paper provides a comprehensive understanding of the background of healthcare LLMs, the evolution and architectural foundation, and the multimodal capabilities. Key methodological aspects—such as domain-specific data acquisition, large-scale pre-training, supervised fine-tuning, prompt engineering, and in-context learning—are explored in the context of healthcare use cases. The paper highlights the trends and categorizes prominent application areas in medicine. Additionally, it critically examines the prevailing technical and social challenges of healthcare LLMs, including issues of model bias, interpretability, ethics, governance, fairness, equity, data privacy, and regulatory compliance. The survey concludes with an outlook on emerging research directions and strategic recommendations for the development and deployment of healthcare LLMs.

## 1. Introduction

Large language models (LLMs) represent a revolutionary advancement in artificial intelligence, demonstrating unprecedented capabilities in understanding and generating human-like text [1]. These models, developed on deep learning and natural language processing technologies, have rapidly integrated into diverse sectors, including healthcare, where they have begun to transform various aspects of medical practice [2]. LLMs excel in processing extensive textual data, deriving insights, and producing high-quality outputs, leading to innovations across clinical decision-making, patient care, medical education, and research.

The healthcare sector, traditionally characterized by large amounts of textual data in the form of medical records, research literature, clinical guidelines, and patient communications, presents a particularly fertile ground for LLM applications [3]. By analyzing and interpreting these complex medical texts, LLMs offer the promise of enhancing diagnostic accuracy, streamlining clinical workflows, improving patient–provider communication, and accelerating medical discoveries [2,4]. The ability of these models to process and synthesize information at unprecedented scales could potentially address critical challenges in healthcare, including information overload, documentation burden, and the need for personalized care [5].

However, the integration of LLMs into healthcare also raises significant concerns regarding data privacy, ethical considerations, factual accuracy, and the potential for bias [4,5]. The high-stakes nature of medical decision-making demands careful validation, responsible deployment, and thoughtful governance of these powerful technologies. As such, understanding both the capabilities and limitations of LLMs in healthcare becomes paramount for researchers, clinicians, policymakers, and technology developers [6].

This comprehensive survey examines the current landscape of LLMs in healthcare, exploring their foundational technologies, methodologies, applications, evaluation frameworks, and challenges. Drawing from recent research across medical informatics, artificial intelligence, and clinical practice, this paper aims to provide a holistic understanding of how LLMs are reshaping healthcare and what future developments may hold. By synthesizing insights from diverse studies and perspectives, this survey seeks to contribute to the responsible and effective integration of LLMs into healthcare systems, ultimately advancing the goal of improved patient outcomes and healthcare delivery [2,5].

## 2. Methodology

### 2.1. Review Design and Rationale

To ensure methodological rigor and transparency, this review was conducted following the Arksey and O’Malley framework [7] for scoping reviews, as refined by Levac et al. [8]. This approach was selected to comprehensively map the breadth of literature on LLMs in healthcare, accommodate heterogeneous study designs, and systematically identify research gaps and future directions. The scoping review methodology is particularly suited to emerging fields such as LLMs in medicine, where the evidence base is rapidly expanding and diverse in scope.

### 2.2. Literature Search Strategy

A systematic literature search was performed across five major databases: PubMed, MEDLINE, IEEE Xplore, ACM Digital Library, Google Scholar, and arXiv. The search covered the period from January 2015 to April 2025, capturing the evolution of transformer-based LLMs and their applications in healthcare. The following Boolean search string was employed: (“large language model” OR “LLM” OR “GPT” OR “transformer model”) AND (“healthcare” OR “medicine” OR “clinical” OR “patient” OR “diagnosis” OR “treatment” OR “medical education”). Searches were limited to English-language publications. Additional records were identified through manual review of reference lists and relevant preprints.

### 2.3. Study Selection Criteria

#### 2.3.1. Inclusion Criteria

- Peer-reviewed articles, conference papers, and preprints focusing on LLM applications in healthcare or medicine.
- Publications from 2015 onward.
- Studies describing, evaluating, or benchmarking LLMs in clinical, educational, research, or administrative healthcare contexts.

#### 2.3.2. Exclusion Criteria

- Non-English publications.
- Studies unrelated to healthcare or lacking sufficient methodological detail.
- Editorials, commentaries, and duplicate records.

### 2.4. Data Extraction and Synthesis

A standardized data extraction form was used to capture key information from each included study. The extracted information encompassed the following elements:

- Source: Refers to the original study or publication cited in the review, providing the foundational reference for the findings presented.
- LLMs Used: Specifies the large language models or AI systems analyzed or implemented in the respective studies (e.g., GPT-4, Med-PaLM, BioGPT, LLaMA, etc.).
- Highlight: Summarizes the core focus or contributions of the study, such as advancements in clinical decision support, diagnostic reasoning, or medical education.
- Application Area: Indicates the specific domain or healthcare context in which the LLMs were applied, such as diagnostics, patient care, clinical decision support, medical education, or drug discovery.

Section 5 provides a detailed summary of key LLMs and their applications in healthcare.

### 2.5. Limitations of the Review

Several limitations should be acknowledged. First, the rapid pace of LLM development means that some recent advances may not be captured. Second, the exclusion of non-English literature may introduce language bias. Third, the diversity of study designs and metrics precluded formal meta-analysis. Finally, most of the included studies originated in high-resource settings, which can limit the generalizability of the findings to global healthcare contexts.

## 3. Background on Large Language Models

### 3.1. Evolution and Architectural Foundations

**Figure 1:**
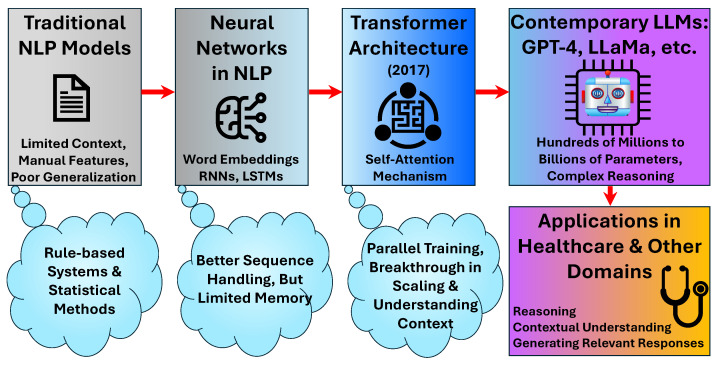
Evolution and architectural foundation of large language models (LLMs).

Large language models have evolved significantly from their predecessors, marking a paradigm shift in natural language processing approaches (Figure 1). Traditional models relied on rule-based systems or statistical methods with limited contextual understanding, whereas modern LLMs leverage deep neural networks, particularly transformer architectures, to process and generate text with remarkable sophistication [4,9]. The transformer architecture, introduced in 2017, revolutionized language modeling through its attention mechanism, enabling models to weigh the importance of different words in context and capture long-range dependencies in text [4].

The evolution of LLMs has been characterized by increasing scale, both in terms of parameter count and training data size. Contemporary models like GPT-4 and LLaMA incorporate billions of parameters, allowing them to capture intricate patterns in language and domain-specific knowledge, including medical terminology and concepts [4]. This scaling has proven crucial for achieving emergent capabilities that smaller models simply cannot manifest, including complex reasoning, nuanced understanding of medical scenarios, and generation of contextually appropriate responses to healthcare queries [5,9].

### 3.2. Foundational Models and Healthcare Adaptation

**Figure 2:**
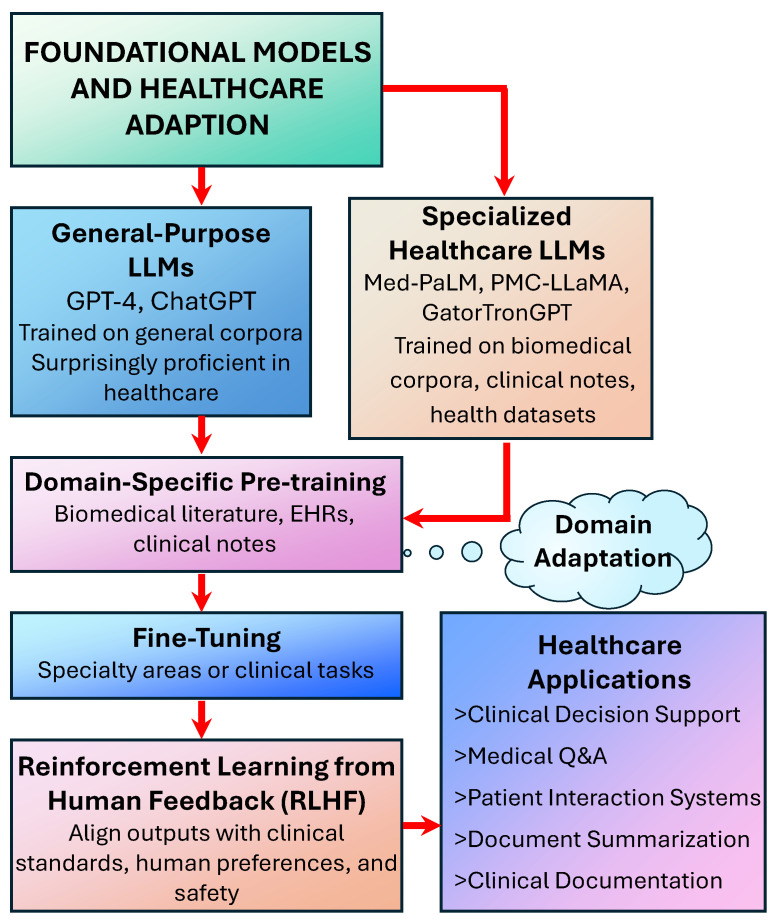
Foundational models and healthcare adaptation of large language models (LLMs).

LLMs in healthcare typically fall into two categories: general-purpose models fine-tuned for medical applications and specialized models developed specifically for healthcare contexts (Figure 2). General-purpose models such as ChatGPT have demonstrated surprising proficiency in medical knowledge despite not being explicitly trained for healthcare applications [6,10]. Meanwhile, specialized models like Med-PaLM, PMC-LLaMA, and GatorTronGPT are designed from the ground up with medical applications in mind, often pre-trained on vast corpora of biomedical literature, clinical notes, and healthcare datasets [4,11].

The adaptation of LLMs for healthcare typically involves domain-specific pre-training on medical corpora, followed by fine-tuning for particular tasks or specialties. This process allows the models to develop robust representations of medical knowledge while calibrating their outputs to align with clinical standards and practices [11]. Furthermore, techniques such as reinforcement learning from human feedback (RLHF) have emerged as critical methods for aligning model outputs with human preferences and medical guidelines, enhancing the relevance and safety of generated content for healthcare applications.

### 3.3. Multimodal Capabilities

Recent advancements have extended LLMs beyond text to incorporate multimodal capabilities, enabling the processing and generation of diverse data types such as images, audio, and structured clinical data [4,5]. This development is particularly significant for healthcare, where diagnostic and treatment decisions often rely on the integration of multiple data modalities, including imaging studies, vital signs, laboratory results, and clinical narratives [5].

**Figure 3:**
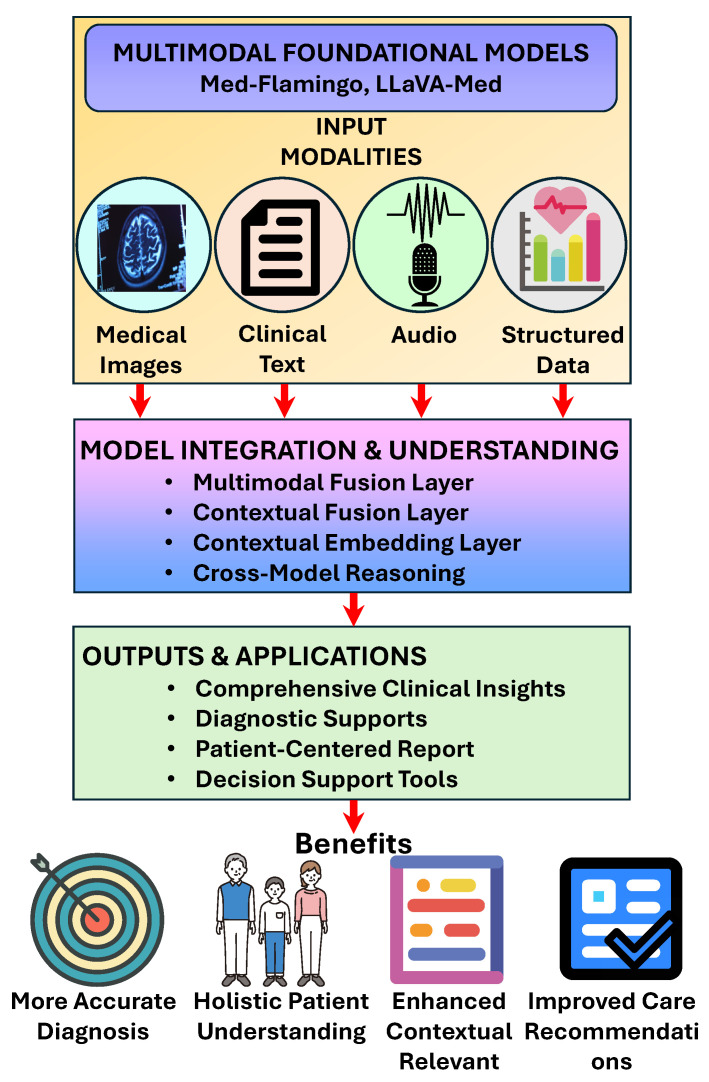
Multimodal capabilities of healthcare large language models in processing and generation of diverse data types such as images, audio, and structured clinical data.

Multimodal LLMs in healthcare can process and interpret various inputs, from radiological images to patient-reported symptoms, providing more comprehensive and contextually informed outputs (Figure 3). For instance, models like Med-Flamingo and LLaVA-Med demonstrate the ability to analyze medical images in conjunction with textual information, potentially enhancing diagnostic accuracy and clinical decision support [11]. This multimodal integration represents a significant advancement toward more holistic and patient-centered applications of artificial intelligence in healthcare settings [5].

## 4. Methodology of LLMs in Healthcare

### 4.1. Data Acquisition and Pre-Training

**Figure 4:**
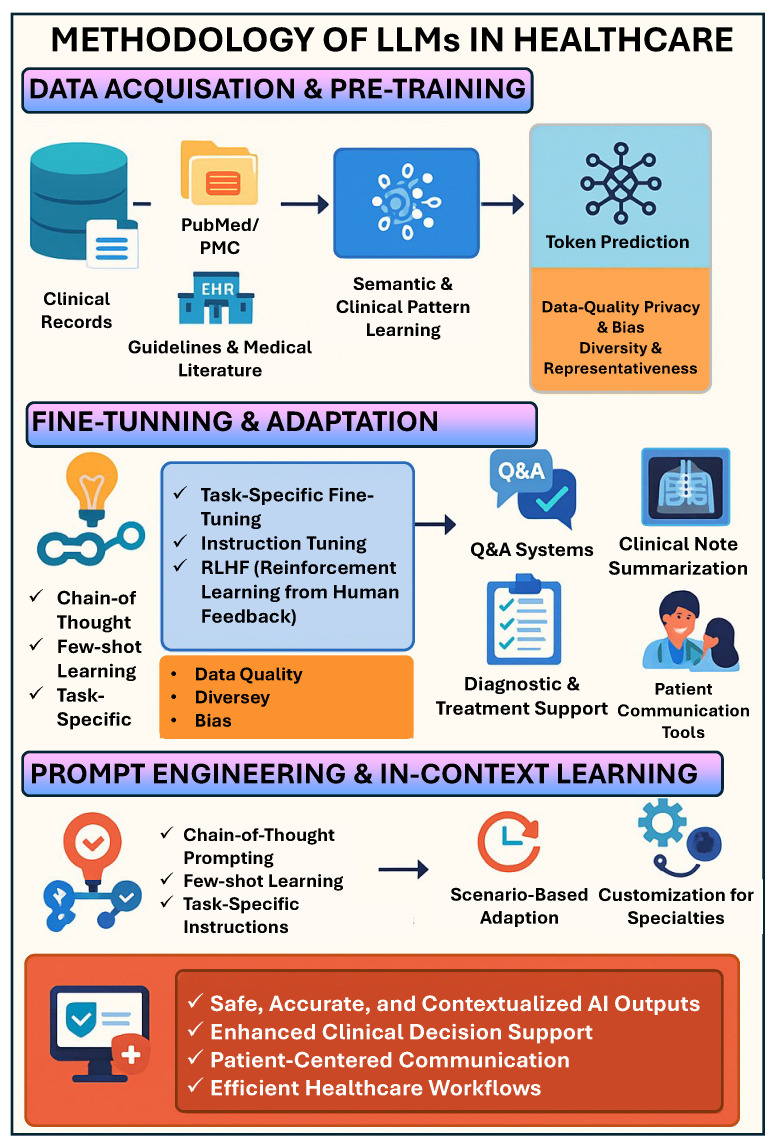
Methodology of LLMs in healthcare: data acquisition and pre-training, fine-tuning and adaptation, prompt engineering, and in-context learning.

The development of effective LLMs for healthcare applications begins with the crucial step of data acquisition. Medical LLMs require vast and diverse datasets encompassing clinical records, medical literature, healthcare guidelines, and domain-specific knowledge resources [11]. These datasets form the foundation upon which models develop their understanding of medical terminology, concepts, and relationships (Figure 4). The quality, diversity, and representativeness of these training data significantly influence the models’ performance and applicability across different healthcare contexts and populations [9,11].

Pre-training involves exposing models to extensive corpora of medical texts to develop general language capabilities and domain-specific understanding. Datasets commonly used for pre-training medical LLMs include PubMed abstracts, PMC Open Access, MIMIC (Medical Information Mart for Intensive Care), and various electronic health record collections [11]. Through pre-training, models learn to predict tokens in context, developing representations of medical language that capture semantic relationships, clinical reasoning patterns, and domain-specific knowledge structures [4].

The scale and diversity of pre-training data present both opportunities and challenges. Although larger and more diverse datasets can improve model performance and generalizability, they also raise concerns regarding data quality, privacy, and the potential incorporation of biases present in historical medical records and literature [4,6]. Consequently, careful curation and preprocessing of training data have emerged as essential considerations in the development of responsible and effective healthcare LLMs [5,11].

### 4.2. Fine-Tuning and Adaptation

Following pre-training, LLMs undergo fine-tuning to adapt their capabilities to specific healthcare tasks, specialties, or use cases. Fine-tuning typically involves additional training on smaller, task-specific datasets with direct supervision for the target application [4]. This process allows models to specialize their outputs for particular clinical contexts while maintaining the broad knowledge base acquired during pre-training.

Task-specific fine-tuning has been applied to adapt LLMs for various healthcare applications, including medical question answering, clinical note summarization, diagnostic assistance, and treatment recommendation [4,11]. For instance, models like Med-PaLM undergo fine-tuning on medical examination questions and clinical vignettes to enhance their performance in diagnostic reasoning and medical knowledge application [11]. Similarly, specialized fine-tuning enables models to generate patient-friendly explanations, summarize clinical literature, or assist in documentation tasks with greater precision and relevance.

Innovative approaches to fine-tuning include instruction tuning, where models learn to follow specific directions relevant to healthcare contexts, and alignment techniques such as reinforcement learning from human feedback (RLHF), which optimize models based on expert evaluations of their outputs. These methods help bridge the gap between the general capabilities of LLMs and the specific requirements of healthcare applications, where precision, safety, and adherence to clinical standards are paramount [4,5].

### 4.3. Prompt Engineering and In-Context Learning

Prompt engineering has emerged as a key methodology for effectively using LLMs in healthcare without requiring extensive model retraining. This approach involves carefully crafting input prompts to guide the model toward generating appropriate, accurate, and clinically relevant outputs [4]. In healthcare contexts, prompts can include patient information, clinical questions, or specific instructions that frame the interaction in ways that leverage the model’s existing knowledge while constraining its responses to align with medical best practices [6].

Advanced prompt engineering techniques, such as chain-of-thought prompting, encourage models to articulate their reasoning processes step by step, mirroring clinical decision-making and making their conclusions more transparent and verifiable [12]. Similarly, few-shot learning through exemplars embedded in prompts allows models to adapt to specific medical tasks by providing demonstrations of desired outputs, enhancing their performance without additional training [4].

In-context learning represents another powerful methodology for healthcare applications, enabling LLMs to adapt to new tasks or domains based on examples provided within the input context [4]. This capability allows healthcare professionals to customize model behavior for specific clinical scenarios, patient populations, or medical specialties by including relevant examples or guidelines in their prompts. The flexibility provided by prompt engineering and in-context learning has facilitated the rapid adaptation of LLMs to various healthcare applications, from the generation of patient education materials to the assistance of complex diagnostic reasoning [12].

## 5. Applications of LLMs in Healthcare

### 5.1. Clinical Decision Support and Diagnostics

Large language models have demonstrated promising capabilities in augmenting clinical decision-making processes across various medical specialties [13,14]. By analyzing patient symptoms, medical records, and relevant data, LLMs can assist healthcare providers in identifying potential diagnoses, suggesting appropriate tests, and recommending evidence-based treatments [4,15,16,17,18,19]. Studies have shown that LLMs can achieve considerable accuracy in diagnostic reasoning, particularly when provided with comprehensive clinical information and well-structured instructions [2,6,15,16]. Specific implementations include systems that analyze symptoms for preliminary diagnosis, models that interpret laboratory results in the clinical context, and tools that identify potential drug interactions or adverse events [5,20]. For instance, Stanford University researchers have employed LLMs to suggest potential treatments for cardiac conditions, while the National Institutes of Health (NIH)’s GatoTron LLM examines electronic health records to detect potential drug interactions [21]. These applications demonstrate how LLMs can serve as cognitive assistants for clinicians, helping them synthesize complex information and consider diverse diagnostic possibilities [10].

Healthcare providers have expressed particular comfort with LLMs functioning in assistive roles, similar to physician extenders or trainees, where they can enhance clinical reasoning while maintaining human oversight of final decisions [10,14]. This alignment with clinician preferences suggests a path for integration that preserves the critical role of human judgment while leveraging the information-processing capabilities of LLMs to enhance diagnostic precision and treatment optimization [2,10,22,23,24].

### 5.2. Medical Education and Training

LLMs are revolutionizing medical education by delivering personalized learning experiences and creating immersive simulations of real-world clinical scenarios. These models can generate customized educational content, adapt to individual learning styles, and provide immediate feedback, enhancing the efficiency and effectiveness of medical training programs [2,25,26,27,28,29]. The ability of LLMs to simulate patient encounters with diverse presentations and complexities offers valuable opportunities for students to develop clinical reasoning skills in a safe and controlled environment [12,30]. Beyond student education, LLMs are proving valuable for continuing professional development, helping practicing clinicians stay up to date with rapidly evolving medical knowledge and guidelines [31,32]. By analyzing the latest research literature and clinical trials, these models can provide concise summaries of emerging evidence, facilitating the integration of new findings into clinical practice [5,28,33]. Furthermore, LLMs have been employed to develop interdisciplinary programs that combine medicine, AI, data analytics, and leadership skills, preparing healthcare professionals for an increasingly digital healthcare landscape [31].

The implementation of LLMs in medical education addresses one of the major challenges in the field: the overwhelming cognitive load faced by students and practitioners [29,34]. By managing the mechanical aspects of information processing, these technologies enable learners to focus on critical thinking, problem-solving, and the humanistic dimensions of medicine, potentially enhancing both technical competence and compassionate care [2].

### 5.3. Patient Care and Communication

LLMs are enhancing patient care through various applications designed to improve provider efficiency, patient engagement, and healthcare accessibility [23,35,36,37]. Virtual medical assistants powered by LLMs can facilitate patient triage, symptom assessment, and care navigation, as exemplified by systems like the NHS’s Florence Chatbot and Babylon Health Chatbot. These tools help direct patients to appropriate levels of care while providing basic health information and addressing common concerns.

The conversational capabilities of LLMs present significant opportunities for bridging patient–provider communication gaps and addressing barriers related to health literacy, language differences, and complex medical terminology [5,38,39]. By translating medical jargon into accessible language, LLMs can help patients better understand their conditions, treatment options, and care plans, potentially improving adherence and outcomes [4,40]. Additionally, these models can assist in obtaining comprehensive patient histories through natural conversation, ensuring thorough documentation while reducing the burden on healthcare providers.

Healthcare providers have recognized the potential of LLMs to enhance patient care through more personalized and efficient service delivery [2,6]. By analyzing individual patient data, medical literature, and clinical guidelines, LLMs can offer tailored insights for diagnosis, treatment planning, and ongoing monitoring, potentially improving patient outcomes, reducing errors, and boosting satisfaction. This personalized approach aligns with the broader movement toward precision medicine, where interventions are customized to individual patient characteristics and preferences [5,6].

### 5.4. Medical Literature Analysis and Research Support

The exponential growth of medical literature presents significant challenges for clinicians and researchers attempting to stay current with the latest evidence [41,42,43]. LLMs are addressing this challenge by efficiently analyzing and summarizing vast volumes of medical literature, helping healthcare professionals maintain awareness of emerging developments and evidence-based practices [4,44]. This capability supports informed clinical decision-making while reducing the time burden associated with literature reviews [4,12].

In research contexts, LLMs are accelerating discovery by analyzing large datasets from medical records, clinical trials, and scientific literature. This analytical power aids in identifying potential new treatments, developing effective therapies, and understanding disease mechanisms through pattern recognition and hypothesis generation [5]. The ability to process and synthesize information across diverse sources enables researchers to identify connections and insights that might otherwise remain obscure [4].

Furthermore, LLMs are streamlining various aspects of the research process, from literature reviews and hypothesis formulation to experimental design and manuscript preparation [2]. By automating routine aspects of research documentation and analysis, these models allow investigators to focus on creative and interpretive aspects of scientific inquiry [5]. This efficiency gain has the potential to accelerate the pace of medical discovery and innovation, ultimately translating to improved patient care and outcomes.

### 5.5. Drug Discovery and Development

The application of LLMs in drug discovery represents a transformative approach to pharmaceutical research, offering the potential to significantly reduce development timelines and costs [4,45,46,47,48]. These models demonstrate remarkable capabilities in analyzing complex molecular structures, identifying promising compounds with therapeutic potential, and predicting the efficacy and safety profiles of candidate drugs [4,49,50,51,52]. By leveraging their pattern recognition abilities, LLMs can suggest novel molecular configurations that might address specific therapeutic targets, potentially expanding the range of treatment options for various conditions [4,51,52,53,54]. Chemical language models specifically designed for pharmaceutical applications have shown notable achievements in de novo drug design [4,53,54]. These specialized models can generate molecular structures with desired properties, predict compound behaviors in biological systems, and optimize candidates for improved pharmacokinetics and reduced side effects [4]. Studies have demonstrated that warm-started models, initialized with pre-trained biochemical language models, outperform baseline approaches in generating high-quality compounds with promising therapeutic potential [4,55]. The integration of LLMs into drug discovery pipelines illustrates the potential for artificial intelligence to transform traditional research and development processes in pharmaceuticals [5,52]. By accelerating the identification and optimization of lead compounds, these models may help address unmet medical needs more rapidly and efficiently, potentially benefiting patients with conditions that currently lack effective treatments [4].

### 5.6. Radiology and Medical Imaging

The integration of LLMs with medical imaging represents a significant advancement in diagnostic capabilities, particularly through multimodal models that can process both visual and textual information [4,11,56,57,58]. By analyzing radiological images in conjunction with clinical data, these systems can assist in the early identification of abnormalities and contribute to more precise diagnostic interpretations [4,58,59,60]. Models such as Med-Flamingo and LLaVA-Med demonstrate the capacity to understand and analyze medical images within their clinical context, potentially enhancing both the efficiency and accuracy of diagnostic processes [11,61,62].

Beyond image interpretation, LLMs are revolutionizing radiological workflow through automated report generation [4,63]. This application addresses the time-consuming and potentially error-prone task of creating detailed radiological reports, especially in high-volume clinical environments [4]. Automated medical report generation from imaging data streamlines the reporting process while maintaining accuracy and comprehensiveness, allowing radiologists to focus on complex cases requiring specialized expertise [4]. Systems like ChatCAD have shown promising results in generating high-quality radiological reports that maintain consistency with human expertise while incorporating relevant clinical information [4].

The advancement of LLMs in radiology could potentially address workforce shortages and improve access to specialized imaging services, particularly in underserved areas [5]. By augmenting the capabilities of radiologists through efficient image analysis and reporting, these technologies may help extend the reach of diagnostic imaging services while maintaining quality and accuracy [4,5]. This application exemplifies how LLMs can enhance existing medical practices rather than replacing human expertise, supporting healthcare providers in delivering more efficient and accessible care [5,10].

### 5.7. Clinical Documentation and Administrative Support

The documentation burden in healthcare represents a significant challenge, with clinicians spending substantial time on administrative tasks that detract from direct patient care [64]. LLMs offer promising solutions by assisting with various aspects of clinical documentation, from generating initial drafts of medical notes to organizing and summarizing patient information [2,65,66]. This capability addresses the dual challenges of ensuring comprehensive documentation while minimizing the time impact on healthcare providers.

Specific applications include automated generation of clinical notes from doctor–patient conversations, standardization of medical notes for improved natural language processing, and organization of clinical data for enhanced accessibility and utility [4]. By streamlining these processes, LLMs can help reduce clinician burnout, improve documentation quality, and allow healthcare providers to dedicate more attention to direct patient interaction [2]. Additionally, these models can assist with coding and billing processes, potentially enhancing revenue cycle management while ensuring compliance with documentation requirements [2].

The efficiency gains offered by LLMs in clinical documentation may have broader implications for healthcare delivery and quality. By reducing the administrative burden on healthcare providers, these technologies could potentially address workforce shortages, improve provider satisfaction, and enhance the overall patient experience through more engaged and attentive care. Furthermore, standardized and comprehensive documentation facilitated by LLMs may support improved clinical research, quality improvement initiatives, and population health management [5].

A summary of the application of LLMs used in healthcare is provided in Table 1 and Table 2.

## 6. Evaluation Frameworks and Benchmarks

### 6.1. Performance Metrics and Assessment Approaches

Evaluating the performance of LLMs in healthcare contexts requires specialized metrics and methodologies that reflect the unique requirements and high-stakes nature of medical applications. Traditional natural language processing metrics such as BLEU and ROUGE are commonly applied to assess the quality of generated text, while task-specific metrics, including accuracy, precision, recall, and AUC, are employed for classification and prediction tasks [4,11]. Additionally, specialized evaluation frameworks like MultiMedQA have been developed to assess the capabilities of LLMs in answering medical questions across various formats, testing both factual accuracy and medical reasoning abilities [11].

Healthcare LLMs are frequently evaluated using established medical benchmarks such as the USMLE (United States Medical Licensing Examination), PubMedQA, and MedMCQA, which provide standardized measures of medical knowledge and reasoning [11]. Performance on these benchmarks serves as an indicator of a model’s capacity to understand and apply medical concepts in clinically relevant contexts [4,11]. More recent evaluation approaches include MMedBench, which covers 21 medical fields and assesses performance across multiple languages, providing deeper insights into model capabilities across diverse healthcare domains and linguistic contexts [11].

The evaluation of multimodal LLMs in healthcare introduces additional complexity, requiring metrics that assess performance across different data types. For image-related tasks, models like PMC-CLIP are evaluated using Recall@K for image–text retrieval and AUC for image classification [11]. Similarly, models like LLaVA-Med and Med-Flamingo undergo evaluation on specialized visual question answering datasets such as VQA-RAD, SLAKE, and Path-VQA to measure their performance in medical imaging applications [11].

### 6.2. Human-Centered Evaluation

While automated metrics provide valuable quantitative assessments, human-centered evaluation approaches play a crucial role in determining the clinical utility and safety of healthcare LLMs [6,10]. Mixed-methods surveys of clinicians have revealed varying levels of comfort with LLM applications in healthcare, with greater acceptance for assistive roles that support rather than replace human decision-making [6]. These findings highlight the importance of incorporating healthcare provider perspectives in the evaluation and implementation of LLMs in clinical settings [6,10].

Expert validation of LLM outputs represents another essential component of evaluation, particularly for applications involving diagnosis, treatment recommendations, or patient communication [6,10]. Studies have employed methodologies ranging from direct comparisons with clinician judgments to more sophisticated approaches where experts assess the quality, accuracy, and safety of model-generated content across different healthcare scenarios [10]. These evaluations provide critical insights into the alignment between LLM outputs and clinical standards, identifying areas where models may require further refinement or human oversight [6,10].

The integration of automated metrics with human judgment offers a more comprehensive evaluation framework for healthcare LLMs [10,11]. For instance, GPT-4 has been used as a reference model for evaluating other LLMs, with its assessments validated against human expert judgments to establish reliability [11]. Similarly, doctors have been involved in comparing responses from different models, providing qualitative assessments that complement quantitative performance metrics [11]. This multifaceted approach to evaluation acknowledges both the technical performance and practical utility of LLMs in healthcare contexts [6,10].

### 6.3. Reproducibility and Validation Challenges

A significant challenge in evaluating healthcare LLMs involves ensuring the reproducibility of results and validating performance across diverse clinical scenarios [6,67]. A systematic review of studies on LLM-based chatbot health advice services revealed considerable variation in reporting quality, with many studies providing insufficient information to identify the specific model being evaluated [67]. This lack of transparency complicates efforts to reproduce findings or compare performance across different studies and implementations [67]. The evaluation of closed-source models presents particular challenges, as researchers often have limited visibility into model architecture, training data, or optimization methods [6,67]. The systematic review found that 99.3% of studies assessed closed-source models without providing adequate information for identification, limiting the scientific value and practical applicability of the evaluations [67]. This opacity in model reporting undermines the ability to build upon existing research or establish reliable benchmarks for healthcare LLM performance [67].

Another validation challenge concerns the ground truth used to define successful performance. The same review found that 64.5% of studies relied on subjective means as the ground truth for evaluating LLM performance, potentially introducing inconsistency and bias into the assessment process [67]. Less than a third of studies addressed the ethical, regulatory, and patient safety implications of clinically integrating LLMs, highlighting a critical gap in comprehensive evaluation frameworks [67]. Addressing these challenges requires more rigorous reporting standards, transparent evaluation methodologies, and greater attention to the practical and ethical dimensions of healthcare LLM implementation [6,67].

### 6.4. Empirical Evaluation and Benchmarking

Recent years have witnessed a surge in quantitative studies evaluating LLMs using established medical benchmarks. For example, Med-PaLM 2 achieved state-of-the-art results on the MultiMedQA suite, including the MedQA (USMLE) benchmark, with accuracy improvements exceeding 19% over previous models. GPT-4 has demonstrated an impressive 93.1% accuracy on MedQA, while models such as BioGPT and Meditron have excelled in biomedical question answering and domain-specific tasks [4,69]. Beyond static benchmarks, real-world validation frameworks such as RWE-LLM have engaged thousands of clinicians across diverse settings, processing over 300,000 clinical interactions and demonstrating robust error detection and safety validation in live environments [70]. These empirical results underscore the growing maturity of LLMs for clinical deployment, while also revealing persistent challenges in generalizability and reliability.

### 6.5. User-Centered and Clinician-Involved Studies

Recognizing that successful integration of LLMs in healthcare requires more than technical excellence, recent research has increasingly incorporated feedback from clinicians and end-users. Human-centered evaluation frameworks, such as the QUEST protocol, involve physicians, nurses, and pharmacists in the systematic assessment of LLM outputs for accuracy, comprehensiveness, bias, and harm in real clinical scenarios [71]. Large-scale studies have shown that clinicians generally view LLMs as valuable adjuncts: improving diagnostic confidence, supporting decision making, and improving workflow efficiency, especially when the models are positioned as assistive tools rather than autonomous decision makers [72,73,74]. For instance, in the RWE-LLM framework, over 6200 licensed clinicians participated in multi-tiered safety validation, providing critical feedback that informed iterative model improvement and ensured alignment with clinical standards [70]. This participatory approach not only enhances model safety but also fosters user trust and acceptance.

## 7. Challenges and Limitations

### 7.1. Data Diversity and Heterogeneity in Healthcare

A central challenge in deploying LLMs in healthcare is the heterogeneity of data across languages, demographic groups, healthcare systems, and data quality. Healthcare data are inherently diverse, encompassing structured records, free-text clinical notes, imaging, and patient-generated information, each with unique formats and standards. This diversity is further complicated by regional differences in language, medical terminology, cultural practices, and health system workflows. For instance, an LLM trained predominantly on English-language data from high-resource environments may underperform in rural or low-resource regions, where local languages, dialects, and unique healthcare practices prevail. Such disparities hinder the generalizability and equity of AI-driven solutions, risking the exacerbation of existing health disparities [75].

Data heterogeneity also includes demographic diversity, age, sex, ethnicity, and socioeconomic status. Models trained on non-representative datasets may perpetuate biases, resulting in less accurate or potentially unsafe recommendations for underrepresented groups [4,75]. Additionally, healthcare data often suffer from varying quality, including missing information, inconsistent coding, and documentation errors, all of which can degrade model reliability and trustworthiness [4,75]. To address these challenges, several strategies are actively pursued:

- Curated and Representative Datasets: Building and utilizing datasets that reflect multiple languages, cultures, and demographic groups is essential. This includes collecting multilingual medical corpora and integrating data from varied healthcare environments [76].
- Domain Adaptation and Fine-Tuning: LLMs can be fine-tuned on region- or institution-specific data to capture local nuances in language and practice, improving model relevance and accuracy for specific settings [76].
- Data Quality Control: Rigorous preprocessing, standardization, and validation protocols enhance data quality and reduce noise or errors in training data [75,76].
- Bias Mitigation Techniques: Algorithmic approaches such as re-sampling, re-weighting, and adversarial training help detect and mitigate biases from unbalanced datasets. Ongoing subgroup evaluation is critical to ensure fairness [4,75].
- Collaborative and Participatory Approaches: Engaging local clinicians, patients, and stakeholders in model development and validation ensures contextual appropriateness and responsiveness to diverse population needs [4,77].

Proactively addressing data heterogeneity is vital for building LLMs that are robust, generalizable, and equitable, ultimately supporting improved health outcomes for all patient groups [4,75].

### 7.2. Technical Challenges and Model Limitations

**Figure 5:**
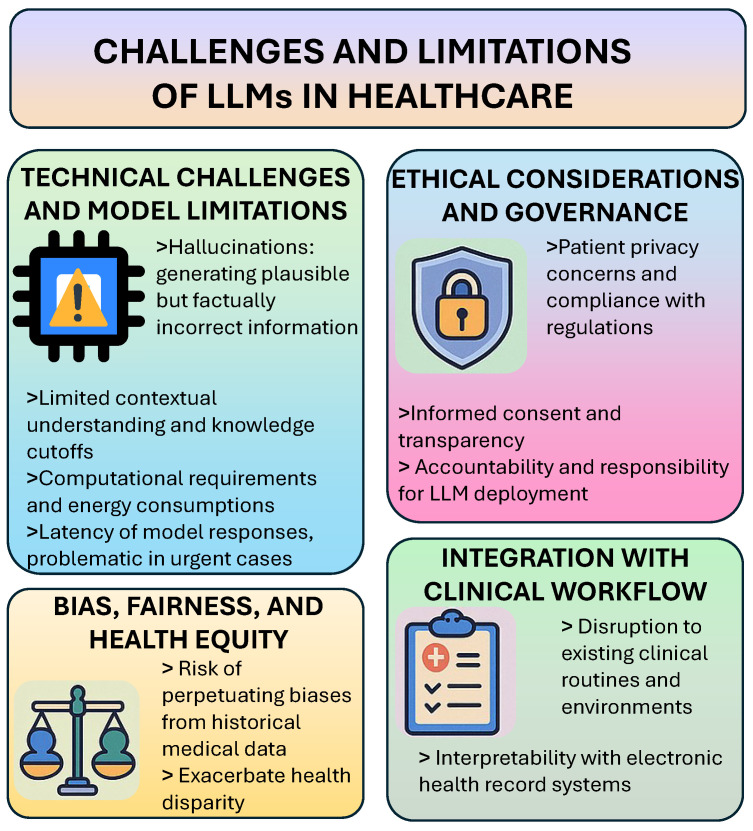
Challenges and limitations of healthcare large language models (LLMs).

Despite their impressive capabilities, LLMs in healthcare face significant technical challenges that limit their immediate clinical utility. Figure 5 presents these challenges and limitations of healthcare LLMs. A primary concern involves hallucination instances where models generate plausible but factually incorrect information—which can have serious consequences in medical contexts where accuracy is paramount [5,6]. This tendency to produce fabricated content, particularly when facing uncertainty or gaps in knowledge, raises concerns about reliability in high-stakes healthcare applications [6].

Limited contextual understanding represents another technical challenge, as current models may struggle to fully comprehend complex medical scenarios or integrate information across different time points or data sources [2,4]. While LLMs can process vast amounts of text, they may miss subtle clinical nuances or fail to appropriately weigh the relevance of different information elements, potentially leading to incorrect or incomplete analyses [2,5]. Additionally, these models typically have knowledge cutoffs based on their training data, limiting their awareness of recent medical developments or emerging health threats [4,6].

The computational requirements of large language models present practical implementation challenges, particularly in resource-constrained healthcare settings [5]. The hardware needed to run sophisticated LLMs may be prohibitively expensive for many healthcare organizations, while the energy consumption associated with model inference raises concerns about environmental sustainability [4,5]. Furthermore, the latency of model responses may be problematic in time-sensitive clinical scenarios where immediate decision support is required [4,5].

### 7.3. Ethical Considerations and Governance

The deployment of LLMs in healthcare raises profound ethical questions that demand careful consideration and robust governance frameworks. Patient privacy represents a fundamental concern, as these models may inadvertently memorize or leak sensitive health information from their training data [6,68]. Ensuring compliance with healthcare privacy regulations such as HIPAA, while leveraging the capabilities of LLMs requires sophisticated data protection measures and appropriate limitations on model inputs and outputs [5,6].

Informed consent and transparency present additional ethical challenges, particularly regarding patient awareness of AI involvement in their care [6]. Patients have the right to understand when LLMs are being used to influence their diagnosis, treatment, or health information, yet conveying this information in an accessible manner without overwhelming individuals remains challenging [6,67]. The potential for patients to develop inappropriate trust in or resistance to LLM-influenced care further complicates these considerations [6].

Accountability and responsibility constitute critical governance concerns, as the distributed nature of LLM development and deployment can obscure lines of liability when errors occur [5,6]. Healthcare organizations, technology developers, regulatory bodies, and individual providers all play roles in ensuring safe and appropriate LLM use, necessitating clear frameworks for oversight, incident reporting, and continuous quality improvement [6,67]. Less than a third of studies address these ethical, regulatory, and patient safety implications, highlighting a significant gap in the current approach to healthcare LLM implementation [67].

### 7.4. Explainability and Interpretability of LLM Outputs

A major barrier to the adoption of LLMs in healthcare is the need for explainability; clinicians and stakeholders must be able to interpret and trust model outputs, especially given the high-stakes nature of medical decision-making [4,78,79]. Transparent and interpretable models foster clinician trust, support shared decision-making, and ensure accountability in patient care [4,77,80,81]. Several methods enhance the interpretability of LLM outputs in healthcare:

- Chain-of-Thought Prompting: This technique encourages models to articulate their reasoning step by step, mirroring clinical decision-making and making conclusions more transparent and verifiable [77].
- Attention Visualization: Visualization tools highlight which parts of the input data the model focused on, providing insights into the decision-making process and identifying potential errors or concerns [78].
- Rule-Based Post Hoc Explanations: Hybrid models that combine LLMs with rule-based systems can generate explanations referencing established clinical guidelines, bridging AI reasoning and human expertise [79,80].
- Uncertainty Quantification: Providing confidence scores or uncertainty estimates alongside outputs allows clinicians to gauge reliability and exercise caution in ambiguous cases [79,81].
- Interactive Interfaces: User-centered designs facilitate interactive exploration of outputs, enabling clinicians to query, challenge, or clarify specific recommendations [79,80].

Explainable AI is not only a technical challenge but also an ethical imperative. As LLMs become more integrated into clinical workflows, ongoing research and development in explainability will be essential for responsible and effective deployment [78,79,80,81].

### 7.5. Bias, Fairness, and Health Equity

LLMs trained on historical medical data risk perpetuating or amplifying existing biases in healthcare, potentially exacerbating health disparities among different demographic groups [4,5]. Studies have demonstrated that certain models exhibit racial bias in patient diagnosis, disproportionately affecting minority groups [5]. This algorithmic bias may result from the underrepresentation of certain populations in training data or the presence of biased clinical practices in the historical record from which models learn [4,6]. Addressing bias requires comprehensive approaches that include diverse and representative training data, careful model evaluation across different demographic groups, and ongoing monitoring for disparate impacts [5,6]. Additionally, the development of debiasing techniques specifically designed for healthcare applications can help mitigate these concerns, though complete elimination of bias remains challenging given the complex socio-historical factors influencing medical data and practice [4,6].

Ensuring equitable access to the benefits of LLM technologies represents another dimension of fairness concerns [5,6]. The digital divide in healthcare, wherein technological advancements disproportionately benefit well-resourced settings and populations, may be exacerbated by LLM integration if deployment primarily occurs in affluent healthcare systems or requires resources unavailable in underserved areas [5]. Thoughtful implementation strategies that prioritize health equity and actively address access disparities are essential for ensuring that LLMs contribute to reducing rather than widening healthcare inequalities [5,6].

### 7.6. Integration with Clinical Workflow

The successful implementation of LLMs in healthcare requires seamless integration with existing clinical workflows, technological infrastructure, and human processes [12]. Disrupting established routines or adding complexity to already busy clinical environments may limit adoption regardless of the potential benefits offered by these technologies [12]. Understanding and adapting to the practical realities of diverse healthcare settings—from large academic medical centers to small community practices—presents a significant implementation challenge [12]. Interoperability with existing electronic health record systems and other healthcare technologies represents a crucial technical aspect of workflow integration [12]. LLMs must be able to access relevant clinical data in real time while maintaining security and privacy, often requiring sophisticated integration solutions that may be difficult to implement across heterogeneous IT environments [5,12]. Furthermore, the outputs of these models need to be presented in formats that align with clinical information needs and decision-making processes, avoiding information overload while providing actionable insights [12].

Healthcare provider acceptance and adaptation constitute human factors that significantly influence successful integration [10,12]. Clinicians may experience resistance to technology-driven changes in practice, particularly when they perceive potential threats to autonomy, increases in workload, or risks to patient care quality [10]. Addressing these concerns through collaborative design approaches, comprehensive training programs, and clear communication about the supportive rather than the replacement role of LLMs can enhance acceptance and appropriate utilization [10,12].

## 8. Future Directions

### 8.1. Multimodal and Domain-Specific Advancements

The future of LLMs in healthcare will likely be characterized by increasingly sophisticated multimodal capabilities, enabling models to process and integrate diverse data types, including text, images, audio, and structured clinical information [4,5]. These advancements will support a more comprehensive analysis of patient cases, potentially enhancing diagnostic accuracy and treatment optimization through holistic data interpretation [5,11]. The development of specialized architectures designed explicitly for multimodal medical data integration represents a promising research direction with significant clinical implications [4,12]. Domain-specific models tailored to particular medical specialties or healthcare contexts will likely proliferate, offering enhanced performance for specialized applications [11,12]. Models designed specifically for fields such as radiology, pathology, mental health, or emergency medicine can incorporate domain-specific knowledge and reasoning patterns, potentially outperforming general-purpose models in these specialized contexts [11,12]. This trend toward specialization may be accompanied by increased transparency in model development and evaluation, addressing current limitations in reproducibility and validation [11,68].

Architectural innovations focused on enhancing the reliability, efficiency, and interpretability of healthcare LLMs represent another important direction for future research [4,5]. Developments may include improved mechanisms for uncertainty quantification, allowing models to express confidence levels in their outputs and appropriately defer to human judgment when facing ambiguity [4,12]. Similarly, advancements in computational efficiency may reduce the resource requirements for deployment, potentially expanding access to these technologies across diverse healthcare settings [4,5].

### 8.2. Human–AI Collaboration Models

The evolution of effective collaboration models between healthcare professionals and LLMs represents a critical area for future development [10,12]. Rather than viewing AI as a replacement for human expertise, research increasingly focuses on creating synergistic relationships where each party contributes complementary strengths [10,12]. These collaborative frameworks may involve dynamic task allocation based on relative capabilities, shared decision-making protocols, and adaptive interfaces that adjust to different clinical scenarios and user preferences [10,12]. Educational approaches for preparing healthcare professionals to work effectively with LLMs constitute another important direction for development [12]. Future medical education may increasingly incorporate training on appropriate AI utilization, critical evaluation of model outputs, and recognition of situations where human judgment should prevail. This educational evolution will help ensure that healthcare providers can leverage the capabilities of LLMs while maintaining the human-centered aspects of care that remain essential to quality healthcare delivery [10].

Implementation science research focused on optimizing the integration of LLMs into clinical practice represents another promising direction [12]. Studies exploring factors that influence successful adoption, identifying best practices for implementation across diverse healthcare settings, and developing metrics for evaluating real-world impact will provide valuable guidance for healthcare organizations seeking to leverage these technologies effectively [12]. This research may help bridge the gap between promising pilot projects and scalable, sustainable implementations that meaningfully improve healthcare delivery.

### 8.3. Regulatory Frameworks and Standard Development

The development of comprehensive regulatory frameworks specifically addressing LLMs in healthcare represents an essential direction for ensuring responsible deployment and patient safety [5,6]. Current regulatory approaches often struggle to address the unique characteristics of these models, including their probabilistic outputs, potential for emergent behaviors, and continuous evolution through additional training or fine-tuning [6,67]. Future regulatory frameworks may incorporate novel approaches such as continuous monitoring requirements, performance thresholds for specific clinical applications, and mandatory reporting of adverse events associated with LLM use [5,6].

Standardization efforts around evaluation methodologies, reporting requirements, and implementation guidelines will likely accelerate as LLMs become more prevalent in healthcare [6,67]. These standards may address current gaps in reproducibility and transparency, potentially requiring more detailed documentation of model characteristics, training data, evaluation procedures, and performance limitations [67]. Additionally, standards for user interfaces, clinical decision support integration, and appropriate disclaimers may help ensure consistent and responsible implementation across different healthcare contexts [6,67]. International coordination on governance approaches represents another important direction, as healthcare LLMs increasingly cross national boundaries through cloud-based deployment models [5,6]. Harmonizing regulatory requirements, ethical standards, and data governance practices across different jurisdictions may help facilitate innovation while maintaining appropriate safeguards [5,6]. This coordination could potentially accelerate the responsible development and deployment of healthcare LLMs by reducing regulatory uncertainty and establishing consistent expectations for developers and implementers [5,6].

### 8.4. Patient-Centered Design and Participatory Approaches

Future developments in healthcare LLMs will likely place greater emphasis on patient-centered design, incorporating patient perspectives, preferences, and needs throughout the development and implementation process [5,6]. Patient involvement in defining use cases, establishing evaluation criteria, and assessing real-world impact can help ensure that these technologies address genuine healthcare needs while respecting patient autonomy and values [6]. This participatory approach may lead to applications that more effectively support shared decision-making, enhance health literacy, and improve patient engagement [5,6].

Personalization capabilities represent another promising direction for patient-centered LLM development [5,6]. Future models may increasingly adapt their interactions based on individual patient characteristics, preferences, and health literacy levels, providing customized information and support that resonates with diverse populations [5]. This personalization could potentially enhance patient understanding, adherence to treatment recommendations, and overall satisfaction with healthcare experiences [5,6]. Accessibility considerations will likely receive greater attention in future healthcare LLM development, ensuring that these technologies benefit populations with diverse needs and abilities [5,6]. This may include multilingual capabilities to serve linguistically diverse communities, interfaces designed for individuals with different levels of digital literacy or physical abilities, and deployment strategies that prioritize underserved populations and healthcare settings [5,6]. Through these patient-centered approaches, healthcare LLMs may contribute to more equitable and inclusive healthcare systems that better serve all populations [5,6].

## 9. Conclusions

This comprehensive survey has examined the rapidly evolving landscape of large language models in healthcare, highlighting their transformative potential while acknowledging the significant challenges that must be addressed for responsible implementation. The application of LLMs across diverse healthcare domains—from clinical decision support and medical education to research acceleration and patient care—demonstrates the remarkable versatility and utility of these technologies in addressing complex healthcare needs [2,4]. At the same time, concerns regarding factual accuracy, privacy, bias, and appropriate integration into clinical workflows underscore the necessity of thoughtful development and governance approaches [2,5,6].

The technical evolution of healthcare LLMs continues at a rapid pace, with advancements in multimodal capabilities, domain specialization, and computational efficiency expanding the range of possible applications [4,11,12]. These developments are complemented by growing attention to evaluation methodologies, regulatory frameworks, and implementation strategies that can help translate promising research into tangible healthcare improvements [6,67]. The emphasis on human–AI collaboration rather than replacement reflects an emerging consensus about the optimal role of these technologies in supporting rather than supplanting human expertise and judgment [10,12].

Looking forward, the responsible integration of LLMs into healthcare systems will require concerted efforts from diverse stakeholders, including technology developers, healthcare providers, regulatory bodies, patient advocates, and researchers [5,6,67]. By addressing current limitations while thoughtfully leveraging the capabilities of these powerful models, the healthcare community has an opportunity to enhance clinical decision-making, improve operational efficiency, accelerate medical discoveries, and ultimately deliver more personalized and effective care to patients [5,6]. As this field continues to evolve, maintaining a balance between innovation and caution will be essential for realizing the full potential of large language models to transform healthcare while upholding the fundamental principles of safety, equity, and patient-centeredness that define quality healthcare delivery [5,6,67].

## Figures and Tables

**Table 1:** Survey of large language models in healthcare applications.

Source	LLMs Used	Highlight	Application Area
M. Johnsen [1]	GPT-4, LLaMA	Overview of foundational LLM concepts and applications	General overview
X. Meng et al. [2]	ChatGPT, GPT-4, Med-PaLM	Comprehensive scoping review of LLM applications in medicine	Medical applications
D. Wang and S. Zhang [3]	ChatGPT, GPT-3.5, GPT-4	Review of applications, advances, and challenges of LLMs in healthcare	Medical and healthcare fields
Z. A. Nazi and W. Peng [4]	ChatGPT, GPT-4, Med-PaLM	Comprehensive review of LLMs in healthcare and medicine	Healthcare and medical domain
K. Zhang et al. [5]	ChatGPT, GPT-4, Med-PaLM	Analysis of transformative impact of LLMs on healthcare	Medicine and healthcare transformation
F. Busch et al. [6]	ChatGPT, GPT-4, Claude	Systematic review of LLM applications and challenges in patient care	Patient care
K. He et al. [9]	ChatGPT, GPT-4, Med-PaLM, Llama-2	Survey focusing on data, technology, applications, accountability and ethics	Healthcare ethics and accountability
M. Spotnitz et al. [10]	ChatGPT, GPT-4, Claude	Survey of clinicians’ perspectives on LLM utility	Clinical utility assessment
D. Zhang et al. [11]	ChatGPT, Med-PaLM, GatorTron	Survey of medical datasets for training and evaluating LLMs	Medical datasets
W. Wang et al. [12]	ChatGPT, GPT-4, Claude, BioMistral	Survey of LLM-based agents in medicine	Medical agents
J. Li et al. [13]	ChatGPT, Med-PaLM, Med-Gemini	Analysis of whether LLMs enhance or replace human expertise	Clinical decision support
M. Yuan et al. [14]	ChatGPT, GPT-4, Claude	Progressive pathway towards AI healthcare assistants	Healthcare assistants
E. Jussupow et al. [15]	Early GPT models, BERT	Investigation of physicians’ decision-making with AI	Medical diagnosis
A. Bojesomo et al. [16]	ChatGPT, GPT-4, Med-PaLM	Systematic review of LLMs for disease diagnosis	Disease diagnosis
P. Karttunen [17]	ChatGPT, GPT-4, Llama-2	Analysis of LLMs for healthcare decision support	Healthcare decision support
I. Almubark [18]	ChatGPT, Med-PaLM, Med-Gemini	Impact of LLMs on disease diagnosis	Disease diagnosis
J. C. L. Ong et al. [19]	ChatGPT, GPT-4, Claude	Development of LLM-based CDSS for medication safety	Medication safety
C. Castaneda et al. [20]	Pre-LLM AI systems	CDSS for diagnostic accuracy and precision medicine	Diagnostic accuracy
X. Yang et al. [21]	GatorTron	Development of LLM specifically for electronic health records	Electronic health records
X. Yang et al. [22]	ChatGPT, Med-PaLM, Med-Gemini	Applications of LLMs in diagnosis and treatment	Disease diagnosis and treatment
K. Holley and M. Mathur [23]	ChatGPT, GPT-4, Claude, Gemini	Exploration of LLMs and generative AI as next frontier in healthcare	Healthcare innovation
B. Yang et al. [24]	DrHouse system	LLM-empowered diagnostic reasoning system using sensor data and expert knowledge	Diagnostic reasoning
B. Santhosh and K. Viswanath [25]	GPT-3.5, GPT-4, BERT	Integration of ML and DL in medical education	Medical education
A. Abd-Alrazaq et al. [26]	ChatGPT, GPT-4, Bard	Opportunities and challenges of LLMs in medical education	Medical education
C. W. Safranek et al. [27]	ChatGPT, GPT-4, Claude	Applications and implications of LLMs in medical education	Medical education
H. C. Lucas et al. [28]	ChatGPT, GPT-4, Med-PaLM	Systematic review of LLMs and implications for medical education	Medical education
T. M. Benítez et al. [29]	ChatGPT, GPT-4, Claude	Promise and pitfalls of LLMs in medical education	Medical education
D. Q. Wang et al. [30]	ChatGPT, Bard, LLaMA	Accelerating integration of ChatGPT into biomedical research	Biomedical research
M. Almansour and F. M. Alfhaid [31]	GPT-4, Claude, Gemini	Personalization of health professional education using generative AI	Health professional education
W. Qian [55]	Early GPT models, BERT	Machine learning applications for drug discovery	Drug discovery

**Table 2:** Continued: Survey of large language models in healthcare applications.

Source	LLMs Used	Highlight	Application Area
D. Domrös-Zoungrana et al. [32]	ChatGPT, GPT-4, Med-PaLM	Considerations for integrating AI in medical education	Medical education
L. Zhui et al. [33]	ChatGPT, GPT-4, Claude	Ethical considerations of LLMs in medical education	Medical education ethics
K. g Lema [34]	GPT-4, Claude, Gemini	AGI applications for medical education and training	Medical education and training
S. Tripathi et al. [35]	ChatGPT, GPT-4, Claude	Optimizing clinical workflow and patient care with LLMs	Clinical workflow optimization
R. Yang et al. [36]	ChatGPT, GPT-4, Llama-2	Development, applications, and challenges of LLMs in healthcare	Healthcare applications
M. ZareiNejad and P. Tavana [37]	ChatGPT, GPT-4, Claude	Applications of generative AI for patient engagement	Patient engagement
Z. Yang et al. [38]	Talk2Care	Facilitating asynchronous patient–provider communication using LLMs	Patient–provider communication
R. Mohammad et al. [39]	Arabic-adapted ChatGPT, Arabic GPT	Optimizing LLMs for Arabic healthcare communication	Multilingual healthcare communication
N. Mannhardt [40]	ChatGPT, GPT-4, Llama-2	Enhancing readability of clinical notes using LLMs	Clinical note comprehension
N. L. Rane et al. [44]	ChatGPT, GPT-4, Bard	Performance of ChatGPT for scientific and research advancements	Medical research
B. Huo et al. [67]	ChatGPT, GPT-4, Claude	Systematic review of LLM chatbots for health advice	Health advice
M. Nydén and D. Bika [45]	ChatGPT, GPT-4, AlphaFold	Medicine design and development in the AI era	Drug development
G. Doron et al. [46]	GPT-4, AlphaFold, ESMFold	Pioneering pharmaceutical R&D with generative AI	Pharmaceutical R&D
S. Harrer et al. [47]	ChatGPT, GPT-4, AlphaFold	AI driving digital transformation in pharmaceutical industry	Pharma transformation
Y. Zhang et al. [48]	ChatGPT, AlphaFold, ESMFold	Accelerating drug discovery and clinical trials with AI	Drug discovery
G. Doron et al. [49]	GPT-4, AlphaFold, Chroma	Driving productivity in pharmaceutical R&D with generative AI	Pharmaceutical productivity
J. Jiang et al. [50]	GPT-4, BERT, RoBERTa	Review of transformer models in drug discovery	Drug discovery
A. Gangwal et al. [51]	GPT-4, ProtGPT2, AlphaFold	Framework, advances, challenges of generative AI in drug discovery	Drug discovery
K. Zhang et al. [52]	ChatGPT, GPT-4, AlphaFold	AI applications in drug development	Drug development
X.h. Liu et al. [53]	ChatGPT, AlphaFold, ESMFold	LLMs facilitating molecular biology and drug development	Molecular biology and drug development
D. Oniani et al. [54]	GPT-4, MolGPT, ChemLLM	Using LLMs for translation between drug molecules and indications	Drug indication mapping
R. AlSaad et al. [56]	GPT-4V, Gemini Pro, Claude 3	Applications and challenges of multimodal LLMs in healthcare	Multimodal healthcare
R. Agbareia et al. [57]	GPT-4V, Med-Flamingo, LLaVA-Med	Quantitative analysis of visual–textual integration in LLMs for diagnosis	Medical diagnosis
R. Guo et al. [58]	Med-Flamingo, LLaVA-Med, PMC-CLIP	Survey of image–text multimodal models in biomedicine	Biomedical imaging
D. Tian et al. [59]	Med-Flamingo, LLaVA-Med, RadBERT	Role of LLMs in medical image processing	Medical image processing
M. Kutbi [60]	GPT-4V, RadBERT, PandaGPT	AI applications for bone fracture detection in medical images	Bone fracture detection
M. Ayaz et al. [61]	MedVLM	Vision–language models for medical applications in consumer devices	Medical image understanding
C. Liu et al. [62]	Med-Flamingo, LLaVA-Med, BioMedCLIP	Foundation models combining visual and language capabilities for medicine	Medical multimodality
N. Soni et al. [63]	ChatCAD, RadGPT, Radiology-GPT	Opportunities and challenges of LLMs in radiology	Radiology
M. A. Rahman [68]	GPT-4V, Gemini Pro, Claude 3	Security and privacy considerations for multimodal LLMs in healthcare	Healthcare security

## Data Availability

No new data were created or analyzed in this study.
